# Danger-Associated Molecular Patterns (DAMPs): Molecular Triggers for Sterile Inflammation in the Liver

**DOI:** 10.3390/ijms19103104

**Published:** 2018-10-10

**Authors:** Sabine Mihm

**Affiliations:** Department of Gastroenterology and Gastrointestinal Oncology, University Medical Center Goettingen, 37075 Goettingen, Germany; smihm@med.uni-goettingen.de; Tel.: +49-551-39-8946

**Keywords:** sterile liver injury, acetaminophen (APAP) intoxication, cholestasis, hepatic ischemia-reperfusion (I/R), non-alcoholic steatohepatitis (NASH), alcoholic liver disease (ALD), danger-associated molecular pattern (DAMP), high mobility group box-1 (HMGB1), type I interferon (IFN)

## Abstract

Inflammatory liver diseases in the absence of pathogens such as intoxication by xenobiotics, cholestatic liver injury, hepatic ischemia-reperfusion injury (I/R), non-alcoholic steatohepatitis (NASH), or alcoholic liver disease (ALD) remain threatening conditions demanding specific therapeutic options. Caused by various different noxae, all these conditions have been recognized to be triggered by danger- or death-associated molecular patterns (DAMPs), discompartmentalized self-structures released by dying cells. These endogenous, ectopic molecules comprise proteins, nucleic acids, adenosine triphosphate (ATP), or mitochondrial compounds, among others. This review resumes the respective modes of their release—passively by necrotic hepatocytes or actively by viable or apoptotic parenchymal cells—and their particular roles in sterile liver pathology. It addresses their sensors and the initial inflammatory responses they provoke. It further addresses a resulting second wave of parenchymal death that might be of different mode, boosting the release of additional, second-line DAMPs. Thus, triggering a more complex and pronounced response. Initial and secondary inflammatory responses comprise the activation of Kupffer cells (KCs), the attraction and activation of monocytes and neutrophil granulocytes, and the induction of type I interferons (IFNs) and their effectors. A thorough understanding of pathophysiology is a prerequisite for identifying rational therapeutic targets.

## 1. Introduction: Danger Self-Patterns in Sterile Liver Disease

Inflammatory liver diseases in the absence of pathogens are regarded “sterile”. Such conditions include the intoxication by xenobiotics (e.g., by para-acetaminophenol, APAP), cholestatic liver injury, hepatic ischemia-reperfusion injury (I/R), non-alcoholic steatohepatitis (NASH), or alcoholic liver disease (ALD). They all keep serious conditions demanding rational therapeutic options, that the thorough understanding of pathophysiology is a prerequisite for. Noxae in sterile liver diseases are various different xenobiotic or even endogenous, biotic molecules. They initiate the release of intracellular compounds either actively or passively from living or dying parenchymal cells. In case of cell death, its mode determines a distinct profile of intracellular molecules that are released into the extracellular space [1]. While necrotic cells are considered to release a bundle of intracellular compounds due to the disruption of plasma membranes in the course of an unregulated or unscheduled disintegration, apoptotic cells largely retain their constituents—packaged into apoptotic bodies—while undergoing a regulated, programmed cell death. Recognition of the fact that these self-compounds are sensed by the host has challenged immunology, the hallmark of which is the discrimination between self and non-self. Polly Matzinger realized that the immune system, beyond, is able to distinguish between self-safe and self-dangerous entities that may arise in pathological conditions [2,3]. These danger- or death-associated molecular patterns (DAMPs) resemble a quite diverse group of discompartmentalized molecules. They comprise nucleic acids in various conformations (e.g., single-/double-stranded (ss/ds) RNA or DNA), nuclear proteins (e.g., high mobility group box-1, HMGB-1), cytosolic proteins (e.g., keratin-18, K18), purine nucleotides (e.g., adenosine triphosphate, ATP), or mitochondrial compounds (e.g., mtDNA, N-formyl peptides), among others. Being sensed by evolutionary conserved pattern recognition receptors (PRRs) [4] within the cytosol, as by nucleotide-binding oligomerization domain (NOD)-like receptors (NLRs) or retinoic acid-inducible gene (RIG)-1-like receptors (RLRs), or from the outside by membranous Toll-like receptors (TLRs) or the receptor for advanced glycation end products (RAGE), DAMPs alert innate immunity responses. Such responses initiate, for instance, the activation of liver resident Kupffer cells (KCs) or dendritic cells (DCs) [5], the mobilization and activation of macrophages, monocytes or neutrophil granulocytes, or the activation of type I interferons (IFN-α/β). It is the host response to these first-line DAMPs that drives sterile liver disease progression. Ultimately, this results in a more marked hepatocellular death, which may be of a different mode than the initial triggering one. This can cause a further increase in a second-line of DAMPs, potentially of different composition, and a more pronounced inflammation. In addition, healing processes might set in and are a challenge for the elucidation of pathophysiology and the identification of therapeutic targets [6]. The relationship between liver cell death and disease activity is evidenced by significant associations of serum alanine aminotransferase (ALT) activity as an indicator for hepatocellular injury and the risk of mortality from liver disease [7,8,9] or all-cause mortality [9,10]. This review summarizes current knowledge of the contribution of first- and of second-line DAMPs in sterile liver disease pathology.

## 2. High Mobility Group Box-1 (HMGB1)

HMGB1 is a highly conserved, abundant, life essential, non-histone nuclear protein expressed in almost all eukaryotic cells [11,12]. It consists of two DNA-binding domains, the N-terminal A-box and the central B-box, and an acidic C-terminal tail. The protein contains two nuclear localization signals. HMGB1 is involved in organizing and stabilizing genomic DNA. Binding less tightly than histones to chromatin, its interaction with nucleosomes and histones loosens packed chromatin and enables its remodeling. Its binding to the minor groove of DNA induces bends in the helical structure, enhancing the interaction of various proteins with DNA and facilitating transcriptional activation. In case of necrotic cell death, nuclear HMGB1 is passively released as a danger pattern [13].

Albeit lacking a signal peptide, HMGB1 also can be actively secreted into the extracellular compartment, e.g., by hepatocytes or by cells of the innate immune system: the acetylation of the two nuclear localization signals neutralizes acidic lysine residues and enables a translocation of acetylated HMGB1 (acHMGB1) from the nucleus to the cytoplasm. From there it is either released in a process of a programmed, pro-inflammatory cell death (pyroptosis) which is dependent on inflammasomes and the activation of caspase-1 [14,15], or via exocytosis of secretory lysosomes [16,17].

Apart from its way of active or passive release, which is mirrored by its acetylation pattern, HMGB1 is able to assemble with various DAMPs as well as with pathogen-associated molecular patterns (PAMPs) such as ssDNA or lipopolysaccharides (LPS), respectively. Either alone or in a complex it interacts with various PRRs as TLR4, TLR9 or RAGE [18,19], thereby contributing to pathology in highly diverse ways [20,21]. For instance, promoting the interaction of nucleic acids and their respective receptors (i.e., TLR9, RAGE) advances a type I IFN response [21].

Moreover, the activity of HMGB1 has been shown to be dependent on the redox status of three cysteine residues: C23 and C45 in the A box domain are able to form an intramolecular disulfide bridge, and C106 in the B box domain. The redox state of HMGB1 released after pyroptosis is generally in the disulfide form, after necrosis in the fully reduced or disulfide forms and after apoptosis in the fully oxidized form [21]. Extracellularly, the fully reduced HMGB1 isoform interacts with the CXC motif chemokine 12 (CXCL12) facilitating its chemoattractant activity, favoring tissue regeneration in liver and muscle [22,23]. The disulfide HMGB1 isoform, in contrast, is recognized as a danger pattern via TLR4 adaptor myeloid differentiation factor-2 (MD-2) promoting inflammation; specific inhibition of binding protects mice against hepatic I/R injury, chemical toxicity, and sepsis [24]. Full oxidation of HMGB1 abrogates its chemokine and cytokine activities and associates with resolution of inflammation [23,25].

### 2.1. HMGB1 in APAP Intoxication: A Passive Release from Dying Hepatocytes Followed by an Active Release from KCs

**Figure 1:**
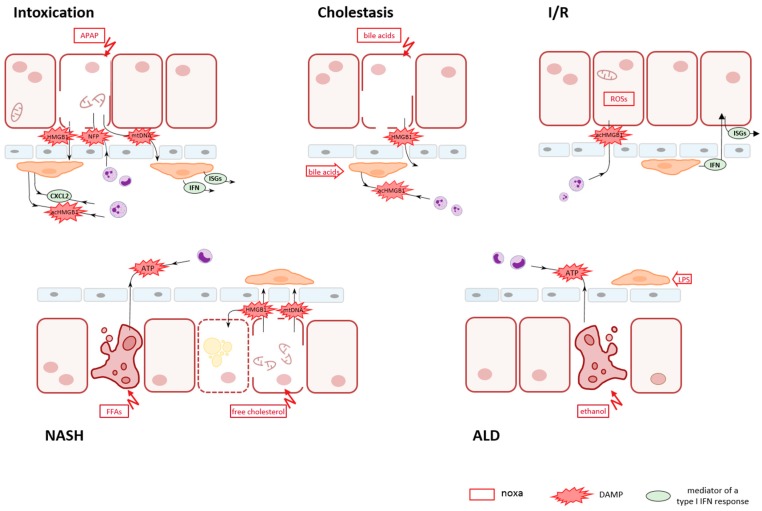
Schematic outline on key events in sterile liver disease conditions. Cell injury of hepatic parenchyma can be provoked by many different noxae such as xenobiotics in intoxication conditions (e.g., APAP), endogenous compounds in cholestatic obstruction (e.g., bile acids) or NASH (e.g., free fatty acids (FFAs), the generation of reactive oxygen species (ROSs) in I/R conditions, or by ethanol in ALD. The release of DAMPs in a first line is common to all conditions. It might be accomplished either actively via secretion from viable or from dying, apoptotic cells or passively via leaking out from necrotic cells. In APAP intoxication, necrotic cell death predominates with the release of HMGB1, mtDNA, and N-formyl peptides (NFP). In cholestasis, bile acids cause a disintegration of hepatocytes, thereby generating a shunt and allowing bile constituents to reach the blood stream and to act also on KCs. In experimental I/R injury, the first insult was demonstrated to be an active secretion of acHMGB1 from intact hepatocytes, while in later stages IFNs become involved in. In the pathogenesis of NASH, concomitant apoptotic and necrotic death of hepatocytes caused by different lipid entities is indicated with the regulated, active release of ATP by apoptotic cells and the passive release of HBMG1 and mtDNA by necrotic cells. In ALD, ethanol-driven apoptosis of parenchymal cells is superposed by microbial products, e.g., LPS, as a result of an impairment of the intestinal mucosa and an increased gut permeability. The first line of DAMPs released by hepatocytes provokes release of a second line of DAMPs and mediators by resident KCs and granulocytes. The composition of second-line DAMPs and mediators governs a secondary innate immune response. This secondary response might comprise cell death of different mode than the initial one, further attraction of neutrophils and monocytes from the circulation, and the induction of an IFN response, thus boosting sterile hepatic inflammation.

APAP intoxication is the leading cause of acute liver injury and acute liver failure in Western countries [26]. It is characterized by a necroinflammatory injury pattern. Specifically, in addition to fulminant necrosis of hepatocytes in the centrolobular areas, activation of KCs occurs as learnt from microscopic examination of liver sections from poisoned patients [27] (Figure 1). In clinical APAP hepatoxicity, quantification of total and of acHMGB1 serum levels have been used as indicators for the extent of necrotic cell death and for the onset of the immune response, respectively [28]. Expectedly, a first rise in total HMGB1 was followed by a timely delayed rise in acHMGB1; and both parameters were found to be associated with a worse outcome [28]. Notably, and of predictive relevance, total HMGB1 release was found to precede the rise in ALT activity [29]. Moreover, two prospective cohort studies recently demonstrated HMGB1 to be qualified as a risk stratification biomarker in paracetamol overdose [30]. The activation of KCs with the secretion of pro-inflammatory cytokines and chemokines resembles a first response to DAMPs that were released by damaged hepatocytes. Experimental evidence for a role of hepatocyte HMGB1 comes from mice with hepatocyte-specific *Hmbg1* knock-out. Subjected to APAP intoxication, HMBG1-deficient mice displayed significantly decreased inflammation and neutrophil granulocyte recruitment when compared to their wild-type counterparts [31,32]. This finding supports data obtained in mice lacking the HMGB1 receptor RAGE on bone marrow-derived cells. Together, data are indicative for an interaction between hepatocyte-derived HMBG1 and KC-derived RAGE resulting in neutrophil-mediated amplification of the initial injury [31,32]. Besides the chemotactic capacity of extracellular HMGB1 and other danger patterns released by necrotic hepatocytes [33], the recruitment of neutrophils is regarded as a secondary response to second-line mediators released by activated KCs. Whether this particular secondary response in APAP intoxication contributes to liver injury or whether this inflammation might be beneficial in terms of the removal of necrotic cell debris and regeneration is a matter of debate as recently reviewed by Wollbright and Jaeschke [34]. Likewise, autophagy was recognized to be involved in pathophysiology of APAP-induced liver injury in an antagonizing, hepatoprotective way [35,36]. The removal of dysfunctional mitochondria (mitophagy) attenuates liver injury [35,37].

### 2.2. HMGB1 in Cholestatic Liver Injury

Similar to the necroinflammatory injury pattern in APAP intoxication, cholestatic liver injury in humans is featured by necrotic cell death (Figure 1). This is supposedly due to lesions in bile duct canaliculi causing a noxious accumulation of bile acids in hepatocytes, and is associated with subsequent sterile inflammatory response [38,39]. In a recent experimental model, bile micro-infarcts were displayed by intravital microscopy [40]. Imaging revealed a compromised mitochondrial membrane potential in hepatocytes followed by rupture of the apical hepatocyte membrane. Apart from their effect on parenchymal cells, these bile infarcts constitute a trans-epithelial shunt between bile canaliculi and sinusoids by which bile constituents leak into blood [40]. In line with in vitro data on human primary hepatocyte cultures exposed to physiological biliary concentrations of human bile acids, measurements on patient samples revealed substantial elevation of native, hypoacetylated HMGB1, serum ALT activities, and a lack of apoptotic biomarkers [41,42]. Because of their amphipathic properties, bile acids were thought to injure the liver directly through their detergent cytolytic effects [43]. More recent findings however point to the initiation of an inflammatory response mediated by the transcription factor Egr1 (early growth response protein 1) and the induction of chemokines, among others, in hepatocytes [43]. In addition to the effects of bile acids on parenchymal cells, bile acids are described to affect and shape macrophage functions [43,44,45,46]. Thus, bile acids may contribute by acting on different target cells to pathophysiology in cholestatic liver diseases. Neutrophil recruitment is likely to be mediated by acHMGB1, which was also found in patient samples. However, other mediators of chemotaxis may also be increased. Neutrophil granulocytes are supposed to be the final executors in cholestatic liver injury [38,43].

### 2.3. HMGB1 in I/R Injury: Early Active Secretion by Hepatocytes

Temporal partial or total obstruction of blood supply and its restoration occur in several disease conditions or during surgical interventions. With regard to the liver, it is encountered during trauma, or unavoidably during surgical procedures as resections or organ transplantation. Ischemia and reperfusion (I/R) injury is characterized by the generation of reactive oxygen species (ROSs). In experimental I/R injury, ROSs have been demonstrated to initiate the active release of acHMGB1 from viable hepatocytes in response to hypoxia [19,47] (Figure 1). This process was shown to be dependent on TLR4 [48]. In an independent investigation, the active release of acHMGB1 from hepatocytes exposed to oxidative stress was shown to be mediated by histone deacetylases, a family of enzymes that remove acetyl groups not only from histones but also from other proteins including HMGB1 [47]. It is hypothesized that histone deacetylases are compromised in activity due to oxidative stress, thus promoting hyperacetylation and subsequent release of acHMGB1, in the absence of necrosis. By employing a transgenic mouse model with conditional *Hmbg1* ablation in epithelial but not bone marrow-derived cells, disulfide HMGB1 was shown to be a key mediator whose absence in epithelial cells correlates with reduced neutrophil recruitment and liver injury [32]. A report showing that hepatocyte-specific genetic deletion of *Hmbg1* increases susceptibility to cellular death in an experimental I/R setting—due to increased nuclear instability—is apparently opposing [49]; differences in genetic deletion strategies may have to be considered.

## 3. Keratin 18 (K18)

Keratin 18 (K18) is the major epithelial-specific intermediate filament protein in the liver, expressed by hepatocytes and cholangiocytes, and its epitopes are specifically recognized by two monoclonal antibodies [50,51]. During apoptosis, K18 is one of the most prominent substrates of activated caspases. Cleavage generates a neo-epitope suitable to discriminate cleaved fragments from the intact protein by a third monoclonal antibody. In the course of apoptotic cell death, caspase-cleaved K18 fragments contribute to the formation of cytoplasmic inclusion bodies, which are released into blood [52,53]. During necrotic cell death in the liver, intact K18 is released into blood, where it circulates within extracellular vesicles [54]. Fragmented K18 and total K18 levels are thus regarded to reflect apoptotic and total cell death, respectively, while the ratio serves as an apoptotic index [54]. K18, so far, raised attention because of its utility in the discrimination of cell death modalities and as a biomarker for liver injury, rather than as a DAMP. This is the reason it is referred to in this context.

### 3.1. NASH: K18 Fragments as an Indicator for Apoptotic Cell Death

NASH is a progressive inflammatory liver disease that develops from non-alcoholic fatty liver, a condition that affects around 25% of the Western and Asian population [55]. Although pathogenesis is yet not well delineated, an altered lipid metabolism with lipid overload in hepatocytes is assumed to contribute to lipotoxicity and to oxidative stress [56,57]. Notably, serum ALT activity is frequently within the normal range and has only limited sensitivity and specificity as a NASH disease marker [58]. Early results showing caspase-cleaved K18 fragments to be increased in patients with NASH when compared to patients with simple steatosis supported apoptotic cell death in NASH. These findings were in agreement with the lack of elevated serum transaminase activities in patients as well as with basic research studies that favor a free fatty acid (FFA)-mediated mito-apoptotic pathogenesis [59] (Figure 1). However, clinical evaluation of the diagnostic performance of K18 fragments is still ambiguous (reviewed in [54]) arguing for additional tissue damaging effector mechanisms.

### 3.2. ALD: Roles for Cleaved and Native K18 as Diagnostic and Prognostic Biomarkers

Similar to NASH, also in ALD serum ALT activity is often only minimally increased. This might be due to an endogenous suppression of the ALT enzyme by ethanol [60]. Likewise, it might point towards apoptosis as the main cell death mode (Figure 1). Serum levels of caspase-cleaved K18 fragments—as indicators for apoptotic cell death—were shown to be associated with disease activity and proposed as a diagnostic marker. In contrast, serum levels of uncleaved K18—used as indicators for necrotic cell death—were described to be associated with mortality. Thus, they are suggested as a prognostic marker in ALD in the longer term [61,62,63].

## 4. Adenosine Triphosphate (ATP)

ATP is a nucleotide that constitutes the main source of energy for most cellular processes. Under physiologic aerobic conditions, it is generated mainly within mitochondria during tricarboxylic acid cycle and respiratory chain and to a lesser extent within the cytoplasm during glycolysis. Through dying cells, ATP is released either passively along with other intracellular contents in the course of necrotic disintegration, or actively in the course of apoptosis. In case of necrosis and also of necroptosis, a cell death mode that is initiated by death receptor ligand binding (as in extrinsic apoptosis) but with a necrotic outcome; however, cellular disintegration is preceded by a mitochondrial impairment and a decline of ATP [64]. In case of apoptosis, release of ATP was shown to be caspase-dependent, not due to leakage of cytoplasmic contents or increased membrane permeability [65], but mediated by plasma membrane channels [66]. Extracellular ATP contributes to tissue homeostasis, as it is chemoattractant for phagocytes (monocytes, macrophages, dendritic cells (DCs), neutrophils) and functions as an endogenous ‘find-me’ signal to the site of apoptotic cells [65]. By acting on ATP-binding purinergic receptors (P2R) on phagocytes, ATP triggers the cytosolic inflammasome danger signal sensing component NLRP3 (pyrin domain containing-3 protein), a NOD-like receptor. The activation of caspase-1, in turn, catalyzes the proteolytic activation of pro-inflammatory interleukin (IL)-1β and IL-18 and facilitates secretion [65,67]. Recruitment and activation of phagocytes by nucleotides is also subject to regulation on different levels: phosphohydrolysis of nucleotides by ectonucleotidases may limit ATP abundance, and nucleoside binding purinergic receptors (P1R) on T effector cells may have opposing, i.e., immunosuppressive, effects [68].

### 4.1. NASH: Confirmatory Evidence for Apoptotic Hepatocellular Death along with Necrotic Cell Death

NASH is featured by an apoptotic cell death of hepatocytes driven by lipotoxic molecules as saturated FFAs or free cholesterol (FC) [59,69,70]. Both of the two fundamental pathways of apoptosis, the extrinsic and the intrinsic one, have been evidenced to contribute to hepatic lipotoxicity in NASH [71]. FFA-induced lipoapoptosis in human hepatocytes has been shown to stimulate the release of ATP, capable of stimulating migration of human monocytes [72,73]. Liver samples from NASH patients present significant increase in inflammasome gene expression (NLRP3, caspase 1, and others) when compared to normal human livers [74] or to liver samples from patients with non-alcoholic fatty liver [75]. These data support the view that ATP released by apoptotic hepatocytes in response to FFAs participates in the recruitment of monocytes in chronic liver injury.

In addition to hepatocyte apoptotic cell death in NASH livers, there is evidence for necrotic cell death as well. Free cholesterol accumulates in human NASH [76,77], and it has been demonstrated to cause hepatocyte apoptotic and necrotic death by activating c-Jun N-terminal kinase 1 (JNK1) [78]. Lipotoxic injury involves mitochondrial permeability transition (MPT) with cytochrome c release, mitochondrial injury, oxidative stress, and ATP depletion culminating in both, apoptosis and necrosis. Secretion of HMGB1 from lipotoxic hepatocytes, moreover, is suggested to facilitate a paracrine cytolytic effect on neighbouring, cholesterol-loaded hepatocytes, which operates via TLR4 [78,79].

### 4.2. ALD: DAMPs and PAMPs

In the pathogenesis of ALD, hepatocyte apoptosis is an early event, too, preceding KC-dependent liver inflammation [80]. Alcohol-induced hepatocyte apoptosis has been shown to be triggered by endoplasmic reticulum (ER) stress involving the ER-resident adaptor STING (stimulator of IFN genes) and the activation of the transcription factor IFN regulatory factor (IRF)-3. STING is a cytosolic PRR for cytosolic DNA. However, while the actual activator of STING remains to be identified, IRF-3 has been shown to be phosphorylated and linking alcohol-induced ER stress with the mitochondrial pathway of hepatocyte apoptosis [80]. In the course of prolonged ethanol exposure, additional processes come into effect and contribute to a more complex pathology. As it applies to the other liver conditions, parenchymal cell death causes inflammation, and alcoholic steatohepatitis is considered to be an enhanced stage of ALD. Additionally, an increased gut permeability by ethanol and a dysbalance of pathogenic and commensal organisms have been recognized as major pathogenic factors in ALD [81]. Both conditions result in higher amounts of LPS, a pathogen pattern of Gram-negative bacteria, in portal and systemic blood of patients with ALD [82,83]. LPS is a potent bacterial endotoxin that is sensed by TLR4 after binding to a co-receptor, CD14, the prototypical PRR. In ALD, recognition of DAMPs from apoptotic hepatocytes thus is superposed by the recognition of PAMPs from gut bacteria. In this regard, ALD does not represent a true sterile inflammatory liver disease.

## 5. Mitochondrial DAMPs

According to the endosymbiont theory of bacteria, mitochondria evolved from energy producing proteobacteria to eukaryote organelles [84,85,86]. Their prokaryotic origin explains structural and molecular similarities with bacteria and a difference from eukaryotes. Like bacteria, mitochondria encode for N-formylated proteins (the initiating amino acid for which is fMet) in contrast to nuclear-encoded proteins. Like the bacterial genome, mitochondrial-derived nucleic acids feature unique structures and properties including (i) a higher proportion of unmethylated CpG dinucleotide motifs different to nuclear DNA, (ii) dsRNA which arises from the bi-directional transcription of the circular genome, (iii) uncapped RNAs, (iv) a three-stranded D-loop structure, an intermediate of mitochondrial DNA (mtDNA) replication, and (v) a higher susceptibility towards oxidation. All these entities—N-formyl peptides (NFPs) and mtDNA with its related species—are recognized as foreign signatures. They are released in the process of ROS-driven mitochondrial membrane permeability transition. During necrosis or necroptosis where plasma membranes are ruptured, mtDAMPs are leaked out into the extracellular space. However, during apoptosis they remain within the cytosol or are packaged into apoptotic bodies.

NFPs are potent chemoattractants acting at the outside, intercellularly. They are sensed by NFP receptors (FPR) which are expressed at high levels on neutrophil granulocytes and mononuclear phagocytes. Ligand binding results in a directed migration to the site of injury, to the production of ROSs and the release of proteolytic enzymes [87,88], fueling a secondary sterile inflammatory response. More specifically, in a hierarchy of directional cues including ATP and the macrophage-derived chemokine CXCL2, NFPs were shown to override other chemotactic signals [89] and to guide neutrophils through non-perfused areas and attract them to sites of sterile inflammation in vivo [90].

MtDNA, released into the cytosol in apoptotic cells, was shown to be sensed by the cyclic GMP-AMP synthase (cGAS), which is a sensor for delocalized, i.e., cytosolic DNA, and which triggers—after interaction with STING—a type I IFN response. This pathway; however, was demonstrated to be suppressed by caspases in the apoptotic cell, thus rendering mitochondrial apoptosis immunological silent [91,92]. Besides, the intracellular release of mtDAMPs by damaged mitochondria is counteracted by autophagy, which is an evolutionarily conserved cellular core process that facilitates the removal of abnormal cellular constituents and organelles [93]. 

MtDNA released into the cytosol of compromised, non-apoptotic cells, in contrast, can effectively engage at least three DNA sensors. MtDNA may trigger pro-inflammatory responses through the activation of NLRP3 inflammasomes and the release of the mature cytokines IL-1β and IL-18. They further may stimulate type I IFN responses through agonizing TLR9. Finally, mtDNA may activate the cGAS-STING pathway resulting in type I IFN induction as well [86]. 

MtDNA released into the extracellular space is sensed by TLR9 from macrophages or neutrophils resulting in the release of pro-inflammatory mediators as tumor necrosis factor alpha (TNF-α) or IL-6 or adhesion molecules [86].

Type I IFNs (IFN-α, IFN-β), in turn, activate transcription of so-called IFN-stimulated genes (ISGs) after binding to their cell surface receptor, IFN-α/β receptor (IFNAR). Signaling takes place via the JAK-STAT(Janus kinase and Signal Transducer and Activator of Transcription proteins) pathway resulting in the formation of the IFN-stimulated gene factor 3 (ISGF3) complex, consisting of phosphorylated STAT1 and STAT2 proteins and IRF-9 [94]. The heterotrimeric transcription factor translocates to the nucleus where it binds to IFN-stimulated regulatory elements (ISRE) in promoter and enhancer regions, the motif ISGs are defined by. Hundreds or approximately 5% of the total number of cellular genes are stimulated by an IFN treatment [94,95,96]. Among them are proteins involved in signal transduction as JAK2, STAT1/2, and IRF-9, able to reinforce the IFN response, and chemokines such as CXCL2 or CXCL10 [96]. ISGs, moreover, comprise PRRs as TLRs, cGAS and STING [97], activation of which enhances sensitizing for PAMPs and DAMPs [94]. Beyond that, two ISGs that are released by macrophages have recently been identified as DAMPs themselves, namely N-myc and STAT interactor (NMI) and IFN-induced protein 35 (IFP35) [98]. So far, roles for ISGs in the pathology of sterile liver diseases beyond just sensing DAMPs as in NASH (via TLR9) and in ALD (via STING), have been reported for APAP intoxication and more particularly for I/R injury (Table 1).

### 5.1. MtDAMPs in APAP Intoxication: A Further Piece of Evidence for a Primarily Necrotic Cell Death and a Role for IFNs

Mitochondrial DAMPs are detectable in the circulation of patients with APAP-induced liver injury [112]. This finding is in line with the evidence outlined above for a primarily necrotic injury pattern with the release of hypoacetylated, nuclear-derived HMGB1 (2.1.). Patients with more mitochondrial damage were shown to be those with a worse prognosis [113], in line with the above-mentioned relationship between liver cell death and mortality in general. Mitochondria are supposed to be central in APAP hepatoxicity in humans [112,113], as APAP-induced hepatotoxicity arises out of the biotransformation of the drug with a depletion of glutathione (GSH) and a subsequent impaired mitochondrial respiration. The resultant decline of ATP levels leads to loss of plasma membrane homeostasis culminating in necrosis. This particular step in the process of cellular disintegration, i.e., cellular swelling as a consequence of rapid decrease in ATP, is also termed oncosis and is thought to contribute to intoxication pathology. In mouse models of APAP intoxication, mtDAMPs have been shown to contribute to neutrophil-mediated injury as blocking the receptors for NFPs (i.e., FPR1) and for mtDNA (TLR9) reduced liver injury [109]. Another group confirmed the release of large amounts of DNA after APAP overdose, its deposition within the liver microvasculature and its lining along the sinusoidal lumen [101]. Furthermore, the authors demonstrated sensing of DNA via cGAS and STING, as mice deficient in cGAS or STING were completely resistant to APAP-induced liver injury. Moreover, by employing IFNAR-deficient mice and in vitro analyses on hepatocytes and non-parenchymal cells they provided evidence for a role of non-parenchymal type I IFNs in amplification of liver injury [101].

In line with the release of mtDNA in a necrotic APAP intoxication pathology, is the finding of the expression of two ISGs. In APAP treated mice, NMI and IFI35 were shown to be released by macrophages into circulation, acting on macrophages through TLR4 and nuclear factor kappa B (NF-κB) pathways inducing pro-inflammatory cytokines [98]. NMI deficiency was shown to reduce liver injury [98].

### 5.2. MtDAMPs in NASH: Evidence for Necrotic Cell Death Too

Liver biopsy specimens from patients with non-alcoholic fatty liver or NASH have been found to feature both structural and functional mitochondrial anomalies [114]. Structural anomalies include organelle enlargement, while functional anomalies eventuate in an enhanced production of ROSs or the accumulation of lipid peroxides [114]. They also feature a higher hepatocyte mitochondria content, and up-regulation of the mitochondrial respiratory capacity is supposed to be a protective adaptation in non-alcoholic fatty livers [115]. As this enhanced respiratory capacity is not detectable in NASH livers, an impaired respiratory capacity coupled to oxidative stress is suggested to be linked to the development into NASH [115].

Furthermore, plasma from patients with NASH was recently shown to contain intact mitochondria and high levels of mitochondrial oxidized DNA enclosed into microparticles of hepatocyte origin [110,116]. Depletion of microparticles from plasma samples derived from obese patients with elevated ALT activities abrogated their ability to activate a TLR9 reporter cell line. By employing a Lysozyme-Cre approach to knock-out TLR9 in myeloid-derived cells, the cell type responsible for TLR9 signaling could be identified as lysozyme-expressing in an in vivo animal model. Extracellular vesicles thus may act as vectors or vehicles to direct the danger pattern from lipid-laden hepatocytes to TLR9- and lysozyme-expressing cells and thus to communicate between injured liver parenchyma and myeloid innate immune cells [110,116,117].

### 5.3. MtDAMPs in I/R Injury: Contribution of IFNs

In I/R injury, the initial release of acHMGB1 by non-compromised hepatocytes is an active process regulated by ROS signaling [19]. It is followed by a second and a third burst of ROSs released by activated KCs and granulocytes, respectively, partly in response to chemoattractant acHMGB1, exacerbating sterile inflammation. This oxidative stress further promotes necrotic cell death with the release of both mitochondrial and nuclear DNA into the cytosol and into circulation.

Consistent with the high abundance of extracellular DNA in I/R injury, IFNs have been shown to contribute to liver I/R injury. IFNAR- but not IFN-γ receptor (IFNGR)-deficient mice failed to elicit inflammatory response and were protected from I/R injury [99]. Particularly, type I IFN signaling was shown to be crucial for mediating synergy between non-parenchymal and parenchymal cells: in response to TLR4 activation, KC-derived type I IFN favors the release of ISGs by hepatocytes, for instance of the chemokine CXCL10.

Likewise, by employing plasmacytoid DC-depleted mice, plasmacytoid DC-derived type I IFN has been shown to promote liver I/R injury by the induction of the ISG IRF-1 from hepatocytes [100]. IRF-1 is known to regulate apoptosis [118,119] and has been shown to enhance hepatic apoptosis in this experimental model as well [100]. IRF-1 indeed has been shown to be up-regulated in human liver allografts after reperfusion [103]. In an orthotopic liver transplantation animal model, IRF-1 deficiency was shown to protect from liver damage [102]. Moreover, IRF-1 has been shown to mediate the initial step in I/R pathogenesis, namely the acetylation and release of HMGB1 from hepatocytes [120]. This and further evidence for the involvement of ISGs, that is a type I IFN response, in hepatic I/R injury is summarized in Table 1.

## 6. Conclusions

To this end, knowledge about pathogenic mechanisms in sterile liver disease conditions is still fragmentary. Evidently, endogenous noxae are identified as well as the primary mode of parenchymal insult they cause. DAMPs, in turn, were demonstrated to trigger a variety of pro-inflammatory processes, the entirety of which appears not yet fully sized. A more thorough understanding of pathophysiology is required to advance rational therapeutic approaches for each of these conditions.

## Figures and Tables

**Table 1:** IFN-regulated genes (ISGs) with evidence for contributing to sterile liver pathology.

General Role	ISG	Specific Role in Sterile Liver Disease		Ref
IFN signaling	JAK2 STAT1/2	IFNAR-deficiency protects from liver injury	I/R	[99]
		IFN-α promotes liver injury	I/R	[100]
		IFNAR-deficiency delays experimental intoxication injury	APAP	[101]
Transcription factors	IRF-1	hepatocellular graft IRF-1 promotes experimental liver transplant injury	I/R	[102]
		promotes experimental liver injury by enhancing hepatocyte apoptosis	I/R	[100]
		promotes injury via IL-15	I/R	[103]
		promotes injury via activation of PMNs	I/R	[104]
		activates autophagic cell death, aggravates hepatic injury	I/R	[105,106]
		mediates HMGB1 release	I/R	[105,106]
	IRF-2	IRF-1 antagonist, protective against liver injury	I/R	[107]
	IRF-3	linking alcohol-induced ER stress with hepatocyte apoptosis	ALD	[80]
	IRF-9	mediator of liver injury	I/R	[108]
Sensitizing for DAMPs	TLR4	involved in ROS-mediated HMGB1 release in liver injury	I/R	[48]
		engages disulfide-HMGB1 thereby promoting liver injury	I/R	[24]
		mediates KC type I IFN activation in experimental liver injury	I/R	[99]
		facilitates HMGB1-driven paracrine cytolytic effect on cholesterol-loaded hepatocytes	NASH	[78,79]
		senses NMI and IFP35 in experimental liver intoxication	APAP	[98]
	TLR9	senses mtDNA in experimental intoxication	APAP	[109]
		promotes experimental liver injury	NASH	[110]
	cGAS	promotes exp. liver injury by sensing extracellular DNA	APAP	[101]
		deletion of cGAS aggravates experimental liver injury independent of STING	I/R	[111]
	STING	promotes exp. liver injury by sensing extracellular DNA	APAP	[101]
		promotes IRF-3-mediated parenchymal apoptotic cell death in response to alcohol	ALD	[80]
Acting as DAMPs	NMI	released by activated macrophages acting on TLR4 in experimental intoxication	APAP	[98]
		NMI deficiency reduces liver injury and mortality	APAP	[98]
	IFP35	released by activated macrophages acting on TLR4 in experimental intoxication	APAP	[98]

## Abbreviations

acHMGB1	acetylated high mobility group box-1
ALT	Alanine aminotransferase
ALD	Alcoholic liver disease
APAP	para-Acetaminophenol
ATP	Adenosine triphosphate
cGAS	cyclic GMP-AMP synthase
CXCL	CXC motif chemokine
DAMP	Danger-associated molecular pattern
DC	Dendritic cell
FFA	Free fatty acid
HMGB1	High mobility group box-1
K18	Keratin-18
IFN	Interferon
IFNAR	IFN-α/β receptor
IFP35	IFN-induced protein 35
IL	interleukin
I/R	Ischemia/reperfusion
IRF	Interferon regulatory factor
ISG	IFN-stimulated gene
KC	Kupffer cell
LPS	Lipopolysaccharide
mtDNA	mitochondrial DNA
NASH	Non-alcoholic liver disease
NFP	N-formyl peptides
NLR	NOD (nucleotide-binding oligomerization domain)-like receptor
NLRP3	pyrin domain containing-3 protein
NMI	N-myc and STAT interactor
NOD	Nucleotide-binding oligomerization domain
P1R	Purinergic receptor, nucleoside binding
P2R	Purinergic receptor, nucleotide binding
PAMP	Pathogen-associated molecular pattern
PRR	Pattern recognition receptor
RLR	RIG-I (retinoic acid-inducible gene-I)-like receptor
ROS	Reactive oxygen species
RAGE	Receptor for advanced glycation endproduct
RIG-1	Retinoic acid-inducible gene-1
STAT	Signal transducer and activator of transcription
STING	Stimulator of interferon genes
TLR	Toll-like receptor

## References

[1] Eguchi A., Wree A., Feldstein A.E. (2014). Biomarkers of liver cell death. J. Hepatol..

[2] Matzinger P. (1994). Tolerance, danger, and the extended family. Annu. Rev. Immunol..

[3] Matzinger P. (2002). The danger model: A renewed sense of self. Science.

[4] Dolasia K., Bisht M.K., Pradhan G., Udgata A., Mukhopadhyay S. (2018). TLRs/NLRs: Shaping the landscape of host immunity. Int. Rev. Immunol..

[5] Almeda-Valdes P., Aguilar Olivos N.E., Barranco-Fragoso B., Uribe M., Mendez-Sanchez N. (2015). The Role of Dendritic Cells in Fibrosis Progression in Nonalcoholic Fatty Liver Disease. BioMed Res. Int..

[6] Woolbright B.L., Jaeschke H. (2015). Sterile inflammation in acute liver injury: Myth or mystery?. Expert Rev. Gastroenterol. Hepatol..

[7] Kim H.C., Nam C.M., Jee S.H., Han K.H., Oh D.K., Suh I. (2004). Normal serum aminotransferase concentration and risk of mortality from liver diseases: Prospective cohort study. BMJ.

[8] Ruhl C.E., Everhart J.E. (2009). Elevated serum alanine aminotransferase and gamma-glutamyltransferase and mortality in the United States population. Gastroenterology.

[9] Lee T.H., Kim W.R., Benson J.T., Therneau T.M., Melton L.J. (2008). Serum aminotransferase activity and mortality risk in a United States community. Hepatology.

[10] Kunutsor S.K., Apekey T.A., Seddoh D., Walley J. (2014). Liver enzymes and risk of all-cause mortality in general populations: A systematic review and meta-analysis. Int. J. Epidemiol..

[11] Lotze M.T., Tracey K.J. (2005). High-mobility group box 1 protein (HMGB1): Nuclear weapon in the immune arsenal. Nat. Rev. Immunol..

[12] Andersson U., Yang H., Harris H. (2018). High-mobility group box 1 protein (HMGB1) operates as an alarmin outside as well as inside cells. Semin. Immunol..

[13] Scaffidi P., Misteli T., Bianchi M.E. (2002). Release of chromatin protein HMGB1 by necrotic cells triggers inflammation. Nature.

[14] Lamkanfi M., Sarkar A., Vande Walle L., Vitari A.C., Amer A.O., Wewers M.D., Tracey K.J., Kanneganti T.D., Dixit V.M. (2010). Inflammasome-dependent release of the alarmin HMGB1 in endotoxemia. J. Immunol..

[15] Lu B., Nakamura T., Inouye K., Li J., Tang Y., Lundback P., Valdes-Ferrer S.I., Olofsson P.S., Kalb T., Roth J. (2012). Novel role of PKR in inflammasome activation and HMGB1 release. Nature.

[16] Bonaldi T., Talamo F., Scaffidi P., Ferrera D., Porto A., Bachi A., Rubartelli A., Agresti A., Bianchi M.E. (2003). Monocytic cells hyperacetylate chromatin protein HMGB1 to redirect it towards secretion. EMBO J..

[17] Gardella S., Andrei C., Ferrera D., Lotti L.V., Torrisi M.R., Bianchi M.E., Rubartelli A. (2002). The nuclear protein HMGB1 is secreted by monocytes via a non-classical, vesicle-mediated secretory pathway. EMBO Rep..

[18] Tian J., Avalos A.M., Mao S.Y., Chen B., Senthil K., Wu H., Parroche P., Drabic S., Golenbock D., Sirois C. (2007). Toll-like receptor 9-dependent activation by DNA-containing immune complexes is mediated by HMGB1 and RAGE. Nat. Immunol..

[19] Tsung A., Sahai R., Tanaka H., Nakao A., Fink M.P., Lotze M.T., Yang H., Li J., Tracey K.J., Geller D.A. (2005). The nuclear factor HMGB1 mediates hepatic injury after murine liver ischemia-reperfusion. J. Exp. Med..

[20] Bianchi M.E. (2009). HMGB1 loves company. J. Leukoc. Biol..

[21] Yang H., Wang H., Chavan S.S., Andersson U. (2015). High Mobility Group Box Protein 1 (HMGB1): The Prototypical Endogenous Danger Molecule. Mol. Med..

[22] Tirone M., Tran N.L., Ceriotti C., Gorzanelli A., Canepari M., Bottinelli R., Raucci A., Di Maggio S., Santiago C., Mellado M. (2018). High mobility group box 1 orchestrates tissue regeneration via CXCR4. J. Exp. Med..

[23] Venereau E., Casalgrandi M., Schiraldi M., Antoine D.J., Cattaneo A., De Marchis F., Liu J., Antonelli A., Preti A., Raeli L. (2012). Mutually exclusive redox forms of HMGB1 promote cell recruitment or proinflammatory cytokine release. J. Exp. Med..

[24] Yang H., Wang H., Ju Z., Ragab A.A., Lundback P., Long W., Valdes-Ferrer S.I., He M., Pribis J.P., Li J. (2015). MD-2 is required for disulfide HMGB1-dependent TLR4 signaling. J. Exp. Med..

[25] Tang D., Billiar T.R., Lotze M.T. (2012). A Janus tale of two active high mobility group box 1 (HMGB1) redox states. Mol. Med..

[26] Ostapowicz G., Fontana R.J., Schiodt F.V., Larson A., Davern T.J., Han S.H., McCashland T.M., Shakil A.O., Hay J.E., Hynan L. (2002). Results of a prospective study of acute liver failure at 17 tertiary care centers in the United States. Ann. Intern. Med..

[27] Hinson J.A., Roberts D.W., James L.P. (2010). Mechanisms of acetaminophen-induced liver necrosis. Handb. Exp. Pharmacol..

[28] Antoine D.J., Jenkins R.E., Dear J.W., Williams D.P., McGill M.R., Sharpe M.R., Craig D.G., Simpson K.J., Jaeschke H., Park B.K. (2012). Molecular forms of HMGB1 and keratin-18 as mechanistic biomarkers for mode of cell death and prognosis during clinical acetaminophen hepatotoxicity. J. Hepatol..

[29] Antoine D.J., Dear J.W., Lewis P.S., Platt V., Coyle J., Masson M., Thanacoody R.H., Gray A.J., Webb D.J., Moggs J.G. (2013). Mechanistic biomarkers provide early and sensitive detection of acetaminophen-induced acute liver injury at first presentation to hospital. Hepatology.

[30] Dear J.W., Clarke J.I., Francis B., Allen L., Wraight J., Shen J., Dargan P.I., Wood D., Cooper J., Thomas S.H.L. (2018). Risk stratification after paracetamol overdose using mechanistic biomarkers: Results from two prospective cohort studies. Lancet Gastroenterol. Hepatol..

[31] Huebener P., Hernandez C., Schwabe R.F. (2015). HMGB1 and injury amplification. Oncotarget.

[32] Huebener P., Pradere J.P., Hernandez C., Gwak G.Y., Caviglia J.M., Mu X., Loike J.D., Jenkins R.E., Antoine D.J., Schwabe R.F. (2015). The HMGB1/RAGE axis triggers neutrophil-mediated injury amplification following necrosis. J. Clin. Investig..

[33] Penzo M., Molteni R., Suda T., Samaniego S., Raucci A., Habiel D.M., Miller F., Jiang H.P., Li J., Pardi R. (2010). Inhibitor of NF-kappa B kinases alpha and beta are both essential for high mobility group box 1-mediated chemotaxis [corrected]. J. Immunol..

[34] Woolbright B.L., Jaeschke H. (2017). Role of the inflammasome in acetaminophen-induced liver injury and acute liver failure. J. Hepatol..

[35] Ni H.M., Bockus A., Boggess N., Jaeschke H., Ding W.X. (2012). Activation of autophagy protects against acetaminophen-induced hepatotoxicity. Hepatology.

[36] Schneider J.L., Cuervo A.M. (2014). Liver autophagy: Much more than just taking out the trash. Nat. Rev. Gastroenterol. Hepatol..

[37] Shan S., Shen Z., Song F. (2018). Autophagy and acetaminophen-induced hepatotoxicity. Arch. Toxicol..

[38] Woolbright B.L., Jaeschke H. (2016). Therapeutic targets for cholestatic liver injury. Expert Opin. Ther. Targets.

[39] Jansen P.L., Ghallab A., Vartak N., Reif R., Schaap F.G., Hampe J., Hengstler J.G. (2017). The ascending pathophysiology of cholestatic liver disease. Hepatology.

[40] Ghallab A., Hofmann U., Sezgin S., Vartak N., Hassan R., Zaza A., Godoy P., Schneider K.M., Guenther G., Ahmed Y.A. (2018). Bile micro-infarcts in cholestasis are initiated by rupture of the apical hepatocyte membrane and cause shunting of bile to sinusoidal blood. Hepatology.

[41] Woolbright B.L., Dorko K., Antoine D.J., Clarke J.I., Gholami P., Li F., Kumer S.C., Schmitt T.M., Forster J., Fan F. (2015). Bile acid-induced necrosis in primary human hepatocytes and in patients with obstructive cholestasis. Toxicol. Appl. Pharmacol..

[42] Woolbright B.L., Antoine D.J., Jenkins R.E., Bajt M.L., Park B.K., Jaeschke H. (2013). Plasma biomarkers of liver injury and inflammation demonstrate a lack of apoptosis during obstructive cholestasis in mice. Toxicol. Appl. Pharmacol..

[43] Li M., Cai S.Y., Boyer J.L. (2017). Mechanisms of bile acid mediated inflammation in the liver. Mol. Asp. Med..

[44] Calmus Y., Poupon R. (2014). Shaping macrophages function and innate immunity by bile acids: Mechanisms and implication in cholestatic liver diseases. Clin. Res. Hepatol. Gastroenterol..

[45] Haselow K., Bode J.G., Wammers M., Ehlting C., Keitel V., Kleinebrecht L., Schupp A.K., Haussinger D., Graf D. (2013). Bile acids PKA-dependently induce a switch of the IL-10/IL-12 ratio and reduce proinflammatory capability of human macrophages. J. Leukoc. Biol..

[46] Sato K., Hall C., Glaser S., Francis H., Meng F., Alpini G. (2016). Pathogenesis of Kupffer Cells in Cholestatic Liver Injury. Am. J. Pathol..

[47] Evankovich J., Cho S.W., Zhang R., Cardinal J., Dhupar R., Zhang L., Klune J.R., Zlotnicki J., Billiar T., Tsung A. (2010). High mobility group box 1 release from hepatocytes during ischemia and reperfusion injury is mediated by decreased histone deacetylase activity. J. Biol. Chem..

[48] Tsung A., Klune J.R., Zhang X., Jeyabalan G., Cao Z., Peng X., Stolz D.B., Geller D.A., Rosengart M.R., Billiar T.R. (2007). HMGB1 release induced by liver ischemia involves Toll-like receptor 4 dependent reactive oxygen species production and calcium-mediated signaling. J. Exp. Med..

[49] Huang H., Nace G.W., McDonald K.A., Tai S., Klune J.R., Rosborough B.R., Ding Q., Loughran P., Zhu X., Beer-Stolz D. (2014). Hepatocyte-specific high-mobility group box 1 deletion worsens the injury in liver ischemia/reperfusion: A role for intracellular high-mobility group box 1 in cellular protection. Hepatology.

[50] Omary M.B., Coulombe P.A., McLean W.H. (2004). Intermediate filament proteins and their associated diseases. N. Engl. J. Med..

[51] Omary M.B., Ku N.O., Strnad P., Hanada S. (2009). Toward unraveling the complexity of simple epithelial keratins in human disease. J. Clin. Investig..

[52] MacFarlane M., Merrison W., Dinsdale D., Cohen G.M. (2000). Active caspases and cleaved cytokeratins are sequestered into cytoplasmic inclusions in TRAIL-induced apoptosis. J. Cell Biol..

[53] Weerasinghe S.V., Ku N.O., Altshuler P.J., Kwan R., Omary M.B. (2014). Mutation of caspase-digestion sites in keratin 18 interferes with filament reorganization, and predisposes to hepatocyte necrosis and loss of membrane integrity. J. Cell Sci..

[54] Ku N.O., Strnad P., Bantel H., Omary M.B. (2016). Keratins: Biomarkers and modulators of apoptotic and necrotic cell death in the liver. Hepatology.

[55] Younossi Z.M., Koenig A.B., Abdelatif D., Fazel Y., Henry L., Wymer M. (2016). Global epidemiology of nonalcoholic fatty liver disease-Meta-analytic assessment of prevalence, incidence, and outcomes. Hepatology.

[56] Rinella M.E. (2015). Nonalcoholic fatty liver disease: A systematic review. JAMA.

[57] Mendez-Sanchez N., Cruz-Ramon V.C., Ramirez-Perez O.L., Hwang J.P., Barranco-Fragoso B., Cordova-Gallardo J. (2018). New Aspects of Lipotoxicity in Nonalcoholic Steatohepatitis. Int. J. Mol. Sci..

[58] Mofrad P., Contos M.J., Haque M., Sargeant C., Fisher R.A., Luketic V.A., Sterling R.K., Shiffman M.L., Stravitz R.T., Sanyal A.J. (2003). Clinical and histologic spectrum of nonalcoholic fatty liver disease associated with normal ALT values. Hepatology.

[59] Feldstein A.E., Canbay A., Angulo P., Taniai M., Burgart L.J., Lindor K.D., Gores G.J. (2003). Hepatocyte apoptosis and fas expression are prominent features of human nonalcoholic steatohepatitis. Gastroenterology.

[60] Woolbright B.L., Jaeschke H. (2018). Is Keratin-18 only a Marker of Cell Death in Acute-On-Chronic Liver Failure?. J. Lab. Precis. Med..

[61] Macdonald S., Andreola F., Bachtiger P., Amoros A., Pavesi M., Mookerjee R., Zheng Y.B., Gronbaek H., Gerbes A.L., Sola E. (2018). Cell death markers in patients with cirrhosis and acute decompensation. Hepatology.

[62] Woolbright B.L., Bridges B.W., Dunn W., Olson J.C., Weinman S.A., Jaeschke H. (2017). Cell Death and Prognosis of Mortality in Alcoholic Hepatitis Patients Using Plasma Keratin-18. Gene Expr..

[63] Bissonnette J., Altamirano J., Devue C., Roux O., Payance A., Lebrec D., Bedossa P., Valla D., Durand F., Ait-Oufella H. (2017). A prospective study of the utility of plasma biomarkers to diagnose alcoholic hepatitis. Hepatology.

[64] Luedde T., Kaplowitz N., Schwabe R.F. (2014). Cell death and cell death responses in liver disease: Mechanisms and clinical relevance. Gastroenterology.

[65] Elliott M.R., Chekeni F.B., Trampont P.C., Lazarowski E.R., Kadl A., Walk S.F., Park D., Woodson R.I., Ostankovich M., Sharma P. (2009). Nucleotides released by apoptotic cells act as a find-me signal to promote phagocytic clearance. Nature.

[66] Chekeni F.B., Elliott M.R., Sandilos J.K., Walk S.F., Kinchen J.M., Lazarowski E.R., Armstrong A.J., Penuela S., Laird D.W., Salvesen G.S. (2010). Pannexin 1 channels mediate ‘find-me’ signal release and membrane permeability during apoptosis. Nature.

[67] Karmakar M., Katsnelson M.A., Dubyak G.R., Pearlman E. (2016). Neutrophil P2X_7_ receptors mediate NLRP3 inflammasome-dependent IL-1beta secretion in response to ATP. Nat. Commun..

[68] Kepp O., Loos F., Liu P., Kroemer G. (2017). Extracellular nucleosides and nucleotides as immunomodulators. Immunol. Rev..

[69] Ioannou G.N. (2016). The Role of Cholesterol in the Pathogenesis of NASH. Trends Endocrinol. Metab..

[70] Neuschwander-Tetri B.A. (2010). Hepatic lipotoxicity and the pathogenesis of nonalcoholic steatohepatitis: The central role of nontriglyceride fatty acid metabolites. Hepatology.

[71] Alkhouri N., Carter-Kent C., Feldstein A.E. (2011). Apoptosis in nonalcoholic fatty liver disease: Diagnostic and therapeutic implications. Expert Rev. Gastroenterol. Hepatol..

[72] Xiao F., Waldrop S.L., Khimji A.K., Kilic G. (2012). Pannexin1 contributes to pathophysiological ATP release in lipoapoptosis induced by saturated free fatty acids in liver cells. Am. J. Physiol. Cell Physiol..

[73] Xiao F., Waldrop S.L., Bronk S.F., Gores G.J., Davis L.S., Kilic G. (2015). Lipoapoptosis induced by saturated free fatty acids stimulates monocyte migration: A novel role for Pannexin1 in liver cells. Purinergic Signal.

[74] Csak T., Ganz M., Pespisa J., Kodys K., Dolganiuc A., Szabo G. (2011). Fatty acid and endotoxin activate inflammasomes in mouse hepatocytes that release danger signals to stimulate immune cells. Hepatology.

[75] Wree A., McGeough M.D., Pena C.A., Schlattjan M., Li H., Inzaugarat M.E., Messer K., Canbay A., Hoffman H.M., Feldstein A.E. (2014). NLRP3 inflammasome activation is required for fibrosis development in NAFLD. J. Mol. Med..

[76] Puri P., Baillie R.A., Wiest M.M., Mirshahi F., Choudhury J., Cheung O., Sargeant C., Contos M.J., Sanyal A.J. (2007). A lipidomic analysis of nonalcoholic fatty liver disease. Hepatology.

[77] Caballero F., Fernandez A., De Lacy A.M., Fernandez-Checa J.C., Caballeria J., Garcia-Ruiz C. (2009). Enhanced free cholesterol, SREBP-2 and StAR expression in human NASH. J. Hepatol..

[78] Gan L.T., Van Rooyen D.M., Koina M.E., McCuskey R.S., Teoh N.C., Farrell G.C. (2014). Hepatocyte free cholesterol lipotoxicity results from JNK1-mediated mitochondrial injury and is HMGB1 and TLR4-dependent. J. Hepatol..

[79] Li L., Chen L., Hu L., Liu Y., Sun H.Y., Tang J., Hou Y.J., Chang Y.X., Tu Q.Q., Feng G.S. (2011). Nuclear factor high-mobility group box1 mediating the activation of Toll-like receptor 4 signaling in hepatocytes in the early stage of nonalcoholic fatty liver disease in mice. Hepatology.

[80] Petrasek J., Iracheta-Vellve A., Csak T., Satishchandran A., Kodys K., Kurt-Jones E.A., Fitzgerald K.A., Szabo G. (2013). STING-IRF3 pathway links endoplasmic reticulum stress with hepatocyte apoptosis in early alcoholic liver disease. Proc. Natl. Acad. Sci. USA.

[81] Szabo G. (2015). Gut-liver axis in alcoholic liver disease. Gastroenterology.

[82] Parlesak A., Schafer C., Schutz T., Bode J.C., Bode C. (2000). Increased intestinal permeability to macromolecules and endotoxemia in patients with chronic alcohol abuse in different stages of alcohol-induced liver disease. J. Hepatol..

[83] Rao R. (2009). Endotoxemia and gut barrier dysfunction in alcoholic liver disease. Hepatology.

[84] Grazioli S., Pugin J. (2018). Mitochondrial Damage-Associated Molecular Patterns: From Inflammatory Signaling to Human Diseases. Front. Immunol..

[85] West A.P., Shadel G.S. (2017). Mitochondrial DNA in innate immune responses and inflammatory pathology. Nat. Rev. Immunol..

[86] Gray M.W., Burger G., Lang B.F. (1999). Mitochondrial evolution. Science.

[87] Panaro M.A., Acquafredda A., Sisto M., Lisi S., Maffione A.B., Mitolo V. (2006). Biological role of the N-formyl peptide receptors. Immunopharmacol. Immunotoxicol..

[88] Gabl M., Sundqvist M., Holdfeldt A., Lind S., Martensson J., Christenson K., Marutani T., Dahlgren C., Mukai H., Forsman H. (2018). Mitocryptides from Human Mitochondrial DNA-Encoded Proteins Activate Neutrophil Formyl Peptide Receptors: Receptor Preference and Signaling Properties. J. Immunol..

[89] Heit B., Robbins S.M., Downey C.M., Guan Z., Colarusso P., Miller B.J., Jirik F.R., Kubes P. (2008). PTEN functions to ‘prioritize’ chemotactic cues and prevent ‘distraction’ in migrating neutrophils. Nat. Immunol..

[90] McDonald B., Pittman K., Menezes G.B., Hirota S.A., Slaba I., Waterhouse C.C., Beck P.L., Muruve D.A., Kubes P. (2010). Intravascular danger signals guide neutrophils to sites of sterile inflammation. Science.

[91] Rongvaux A., Jackson R., Harman C.C., Li T., West A.P., de Zoete M.R., Wu Y., Yordy B., Lakhani S.A., Kuan C.Y. (2014). Apoptotic caspases prevent the induction of type I interferons by mitochondrial DNA. Cell.

[92] White M.J., McArthur K., Metcalf D., Lane R.M., Cambier J.C., Herold M.J., van Delft M.F., Bedoui S., Lessene G., Ritchie M.E. (2014). Apoptotic caspases suppress mtDNA-induced STING-mediated type I IFN production. Cell.

[93] Nakahira K., Haspel J.A., Rathinam V.A., Lee S.J., Dolinay T., Lam H.C., Englert J.A., Rabinovitch M., Cernadas M., Kim H.P. (2011). Autophagy proteins regulate innate immune responses by inhibiting the release of mitochondrial DNA mediated by the NALP3 inflammasome. Nat. Immunol..

[94] Schneider W.M., Chevillotte M.D., Rice C.M. (2014). Interferon-stimulated genes: A complex web of host defenses. Annu. Rev. Immunol..

[95] Stark G.R., Darnell J.E. (2012). The JAK-STAT pathway at twenty. Immunity.

[96] Grunvogel O., Esser-Nobis K., Windisch M.P., Frese M., Trippler M., Bartenschlager R., Lohmann V., Binder M. (2016). Type I and type II interferon responses in two human liver cell lines (Huh-7 and HuH6). Genom. Data.

[97] Ma F., Li B., Yu Y., Iyer S.S., Sun M., Cheng G. (2015). Positive feedback regulation of type I interferon by the interferon-stimulated gene STING. EMBO Rep..

[98] Xiahou Z., Wang X., Shen J., Zhu X., Xu F., Hu R., Guo D., Li H., Tian Y., Liu Y. (2017). NMI and IFP35 serve as proinflammatory DAMPs during cellular infection and injury. Nat. Commun..

[99] Zhai Y., Qiao B., Gao F., Shen X., Vardanian A., Busuttil R.W., Kupiec-Weglinski J.W. (2008). Type I, but not type II, interferon is critical in liver injury induced after ischemia and reperfusion. Hepatology.

[100] Castellaneta A., Yoshida O., Kimura S., Yokota S., Geller D.A., Murase N., Thomson A.W. (2014). Plasmacytoid dendritic cell-derived IFN-alpha promotes murine liver ischemia/reperfusion injury by induction of hepatocyte IRF-1. Hepatology.

[101] Araujo A.M., Antunes M.M., Mattos M.S., Diniz A.B., Alvarenga D.M., Nakagaki B.N., Carvalho E., Lacerda V.A.S., Carvalho-Gontijo R., Goulart J. (2018). Liver Immune Cells Release Type 1 Interferon Due to DNA Sensing and Amplify Liver Injury from Acetaminophen Overdose. Cells.

[102] Ueki S., Dhupar R., Cardinal J., Tsung A., Yoshida J., Ozaki K.S., Klune J.R., Murase N., Geller D.A. (2010). Critical role of interferon regulatory factor-1 in murine liver transplant ischemia reperfusion injury. Hepatology.

[103] Yokota S., Yoshida O., Dou L., Spadaro A.V., Isse K., Ross M.A., Stolz D.B., Kimura S., Du Q., Demetris A.J. (2015). IRF-1 promotes liver transplant ischemia/reperfusion injury via hepatocyte IL-15/IL-15Ralpha production. J. Immunol..

[104] Yang M.Q., Du Q., Goswami J., Varley P.R., Chen B., Wang R.H., Morelli A.E., Stolz D.B., Billiar T.R., Li J. (2018). Interferon regulatory factor 1-Rab27a regulated extracellular vesicles promote liver ischemia/reperfusion injury. Hepatology.

[105] Yu Y., Li S., Wang Z., He J., Ding Y., Zhang H., Yu W., Shi Y., Cui Z., Wang X. (2017). Interferon regulatory factor-1 activates autophagy to aggravate hepatic ischemia-reperfusion injury via the P38/P62 pathway in mice. Sci. Rep..

[106] Cui Z., Li S., Liu Z., Zhang Y., Zhang H. (2018). Interferon Regulatory Factor 1 Activates Autophagy to Aggravate Hepatic Ischemia-Reperfusion Injury by Increasing High Mobility Group Box 1 Release. Cell. Physiol. Biochem..

[107] Klune J.R., Dhupar R., Kimura S., Ueki S., Cardinal J., Nakao A., Nace G., Evankovich J., Murase N., Tsung A. (2012). Interferon regulatory factor-2 is protective against hepatic ischemia-reperfusion injury. Am. J. Physiol. Gastrointest. Liver Physiol..

[108] Wang P.X., Zhang R., Huang L., Zhu L.H., Jiang D.S., Chen H.Z., Zhang Y., Tian S., Zhang X.F., Zhang X.D. (2015). Interferon regulatory factor 9 is a key mediator of hepatic ischemia/reperfusion injury. J. Hepatol..

[109] Marques P.E., Amaral S.S., Pires D.A., Nogueira L.L., Soriani F.M., Lima B.H., Lopes G.A., Russo R.C., Avila T.V., Melgaco J.G. (2012). Chemokines and mitochondrial products activate neutrophils to amplify organ injury during mouse acute liver failure. Hepatology.

[110] Garcia-Martinez I., Santoro N., Chen Y., Hoque R., Ouyang X., Caprio S., Shlomchik M.J., Coffman R.L., Candia A., Mehal W.Z. (2016). Hepatocyte mitochondrial DNA drives nonalcoholic steatohepatitis by activation of TLR9. J. Clin. Investig..

[111] Lei Z., Deng M., Yi Z., Sun Q., Shapiro R.A., Xu H., Li T., Loughran P.A., Griepentrog J.E., Huang H. (2018). cGAS-mediated autophagy protects the liver from ischemia-reperfusion injury independently of STING. Am. J. Physiol. Gastrointest. Liver Physiol..

[112] McGill M.R., Sharpe M.R., Williams C.D., Taha M., Curry S.C., Jaeschke H. (2012). The mechanism underlying acetaminophen-induced hepatotoxicity in humans and mice involves mitochondrial damage and nuclear DNA fragmentation. J. Clin. Investig..

[113] McGill M.R., Staggs V.S., Sharpe M.R., Lee W.M., Jaeschke H., Acute Liver Failure Study Group (2014). Serum mitochondrial biomarkers and damage-associated molecular patterns are higher in acetaminophen overdose patients with poor outcome. Hepatology.

[114] Begriche K., Massart J., Robin M.A., Bonnet F., Fromenty B. (2013). Mitochondrial adaptations and dysfunctions in nonalcoholic fatty liver disease. Hepatology.

[115] Koliaki C., Szendroedi J., Kaul K., Jelenik T., Nowotny P., Jankowiak F., Herder C., Carstensen M., Krausch M., Knoefel W.T. (2015). Adaptation of hepatic mitochondrial function in humans with non-alcoholic fatty liver is lost in steatohepatitis. Cell Metab..

[116] Handa P., Vemulakonda A., Kowdley K.V., Uribe M., Mendez-Sanchez N. (2016). Mitochondrial DNA from hepatocytes as a ligand for TLR9: Drivers of nonalcoholic steatohepatitis?. World J. Gastroenterol..

[117] Inzaugarat M.E., Wree A., Feldstein A.E. (2016). Hepatocyte mitochondrial DNA released in microparticles and toll-like receptor 9 activation: A link between lipotoxicity and inflammation during nonalcoholic steatohepatitis. Hepatology.

[118] Kano A., Haruyama T., Akaike T., Watanabe Y. (1999). IRF-1 is an essential mediator in IFN-gamma-induced cell cycle arrest and apoptosis of primary cultured hepatocytes. Biochem. Biophys. Res. Commun..

[119] Savitsky D., Tamura T., Yanai H., Taniguchi T. (2010). Regulation of immunity and oncogenesis by the IRF transcription factor family. Cancer Immunol. Immunother..

[120] Dhupar R., Klune J.R., Evankovich J., Cardinal J., Zhang M., Ross M., Murase N., Geller D.A., Billiar T.R., Tsung A. (2011). Interferon regulatory factor 1 mediates acetylation and release of high mobility group box 1 from hepatocytes during murine liver ischemia-reperfusion injury. Shock.

